# Influence of the Nutrients on the Biomass and Pigment Production of *Chlorociboria aeruginascens*

**DOI:** 10.3390/jof5020040

**Published:** 2019-05-16

**Authors:** Stephanie Stange, Susanne Steudler, Hubertus Delenk, Anett Werner, Thomas Walther, André Wagenführ

**Affiliations:** 1 Faculty of Mechanical Science and Engineering, Institute of Natural Materials Technology, Technische Universität Dresden, Chair of Wood Technology and Fibre Materials Technology, 01069 Dresden, Germany; hubertus.delenk@tu-dresden.de (H.D.); andre.wagenfuehr@tu-dresden.de (A.W.); 2 Faculty of Mechanical Science and Engineering, Institute of Natural Materials Technology, Technische Universität Dresden, Chair of Bioprocess Engineering, 01069 Dresden, Germany; susanne.steudler@tu-dresden.de (S.S.); anett.werner@tu-dresden.de (A.W.); thomas_walther@tu-dresden.de (T.W.)

**Keywords:** *Chlorociboria aeruginascens*, xylindein, fungal pigments, fungal growth conditions, fungal polyketides, nitrogen limitation

## Abstract

The blue-green pigment xylindein, produced by the soft rot fungus *Chlorociboria aeruginascens*, is of considerable interest for various applications such as the veneer industry or organic semiconductors. The studies presented were performed in order to understand the fungal growth as well as the pigment production of *C. aeruginascens*. Therefore, various nutrient compositions were investigated. As a result, observations of the formation of xylindein through *C. aeruginascens* decoupling from growth were made. In the primary metabolism the uncolored biomass is formed. Various carbohydrates were determined as nutrients for the fungus and as a nitrogen source it was observed that the fungus prefers the complex organic nitrogen source, that being yeast extract. Furthermore, it was discovered that the ratio between carbohydrate and nitrogen sources encourages the switch of the metabolism and therewith the production of the blue-green pigment xylindein.

## 1. Introduction

Fungi of the genus *Chlorociboria* sp. cause a special blue-green discoloration in wood. Wood like this was used in many historical intarsias during the 15th century [1] and it is still possible to admire this historic blue-green colored wood e.g., at the choir stalls of the Nikolaus-Gaetke-chapel in the St. Elizabeth’s Church in Wrocław (Poland) that was built in 1590. The fungi *Chlorociboria* sp. still piques the interest of scientists but also of craftsmen due to its color and wood spalting behavior.

The small cup-shaped fruit bodies of *Chlorociboria aeruginascens* (green elfcup) have a size from 2.5 cm up to 5 cm [2]. The fruit bodies are completely colored in a blue-green color. *C. aeruginascens* belongs to the group of Ascomycota and forms eight unicellular ascospores [3]. The spores are surrounded by small blue-green pigmented vesicles of up to 0.2 µm, when they are released [4].

Concerning its growth habits on wood, *Chlorociboria* sp. belongs to the soft rot fungi. For this reason, higher moisture contents are preferred by this fungus. The mycelium is usually white and changes its color to blue-green under certain growth conditions. The fungal pigment xylindein is responsible for the color of the fruit bodies as well as the discoloration of infected wood. The blue-green pigmentation in wood is predominantly in sapwood while the pigment itself is located in the fungal mycelium and after a certain cultivation time it can diffuse into the growth media [5].

The controlled cultivation and additionally, the biotechnological production of xylindein is relevant for further fields of application. Boonloed et al. [6] and Giesbers et al. [7] describe its potential as a fluorescent labeling agent as well as in organic semiconductor applications. The wood industry, especially the veneer producers, are looking for new wood modification options for example, spalting and therefore, *Chlorociboria* sp. is also of interest [8]. Consideration might be to inoculate wood with *Chlorociboria* sp., but the inoculum must have a sufficient amount in order to prevent contamination, which is only producible with biotechnological process engineering [9,10]. The focus of this study was not to determine the wood spalting process with *Chlorociboria* sp., but rather the growth behavior as well as the pigmentation of the fungus as a preparation for biotechnological cultivation on larger scales.

From a chemical perspective, xylindein is an extended quinone [11,12]. There have been several unsuccessful attempts to synthesize xylindein [11,13]. The provision of xylindein is created these days by extraction with organic solvents such as dichloromethane (DCM) or hot chloroform out of blue-green *Chlorociboria* sp.-culture, colored culture media, fruit bodies, and even naturally infected discolored wood [14,15,16]. Due to its high toxicity, the use of DCM as a solvent is limited to utilization in small amounts.

Xylindein is produced as a secondary metabolite by *Chlorociboria* sp. and it is not essential for fungal growth. Its biomass as well as its pigment production depends extremely on the fungal-available nutrients [9,17,18]. Frenzel [18] carried out the first experiments with *Chlorociboria aeruginosum* focusing on the influence of nutrients on the color of the mycelium at the beginning of the 20th century. He was using agar based and gelatin based media with different carbon and nitrogen sources as well as protein sources. The conclusions drawn by his studies are that hard wood just like beech, birch, maple and oak as well as organic growth media made of plum or malt extract and inorganic nutrients mixed with glucose or different disaccharides support the growth and the pigment production of *Chlorociboria aeruginosum* [18]. Additionally, Robinson et al. [17] and Tudor et al. [4] examined the growth habits of *Chlorociboria* sp., while they were using petri dishes with malt extract agar based media mixed with different wood chips made of sugar maple [4,17], aspen [4,17], tree of heaven [17] and previously spalted aspen [17]. The best results concerning growth and pigmentation were observed in malt extract agar mixed with wood chips from sugar maple and previously spalted aspen. Our own investigations have shown comparable results using agar-agar mixed with the bark of oak, birch, poplar and plane tree as well as rice extract agar [9].

In addition to the cultivation on solid media, fungi can also be cultivated as liquid cultures. In our own investigations [9], 12-well plates have been prepared with various nutrient broths in different concentrations inter alia with standard media, which are known for fungal cultivation, e.g., Kirk media and complex media, e.g., rice extract, wood extracts, various fruit juices or consommé. Whereby the complex media, especially the fruit juices, have shown the best results according to growth and pigment production [9]. The positive effect of the determined nutrients was confirmed in shaking flasks (250 mL volume). The liquid cultivation of *Chlorociboria* sp. was also carried out by the work group around Robinson. For their investigation, they used 2% malt extract [5]. Weber et al. [5] describe the positive cultivation with a maxima pigment yield after 28 days of cultivation. The xylindein production and the cultivation of *Chlorociboria* sp. have only been implemented on a laboratory scale until now.

The fungal-available nutrients have a certain influence on the biomass and pigment production besides it being a successful process strategy. Thus, *Chlorociboria* sp. is currently cultivated in complex media e.g., orange juice or malt extract as given in the literature and their natural variability have a certain influence on the repeatability of the experiments, one objective of this work is to replace such media. Consequently, the main aims of the work presented here are to increase the biomass of *C. aeruginascens* for shorter cultivation times and accordingly to increase the yield of xylindein by determining the optimal cultivation medium and analyzing the growth and product building habits of *C. aeruginascens*. Influences of environmental growth factors on the biomass and pigment production of *C. aeruginascens* are also tested and described in Stange et al. [19].

## 2. Materials and Methods

For all the experiments presented, the fungus *Chlorociboria aeruginascens* IHIA39 (of the International Institute (IHI) Zittau) was used. The fungus was maintained on orange juice-agar plates (50% orange juice from Sonniger^®^, 100% fruit content, manufactured for ALDI Nord, Essen, Germany; 30 g/L Agar-Agar bacteriological from Carl Roth GmbH & Co. KG, Karlsruhe, Germany) or in a 5% orange juice medium (in shaking flasks at 160 rpm) [9]. The fungus was cultivated at 22 °C. All inoculation cultures had an age of 7 days before transferring into the experimental setup. The used 5% orange juice medium was analyzed concerning the main ingredients. It contains the carbohydrates glucose (1.54 ± 0.2 g/L), sucrose (0.94 ± 0.1 g/L) and fructose (1.78 ± 0.1 g/L). The total concentration of reduced sugar amounts to 3.8 ± 0.04 g/L. The total nitrogen content was measured to 39.60 ± 0.1 mg_N_/L and the organic acids was analyzed to 320.5 ± 16.2 mg/L. The used orange juice has a natural pH-value of 4.

### 2.1. Influence of Different Carbon and Nitrogen Sources on the Growth of Chlorociboria aeruginascens 

To determine the usable nitrogen and carbohydrate sources for *C. aeruginascens,* 12-well plates (Cellstar^®^, Greiner bio-one, Kremsmünster, Austria) with a work volume of 3 mL were prepared with 0.1 g/L nitrogen source (ammonium chloride equate 26 mg_N_/L, yeast extract equate 8 mg_N_/L, nicotinic acid equate 11 mg_N_/L) and 1 g/L carbohydrate source (d-arabinose, d-fructose, d-galactose, d-glucose, d-maltose, d-mannose, d-sucrose, d-xylose) as well as a mineral solution (0.5 g/L KH_2_PO_4_; 0.1 g/L MgSO_4_·7 H_2_O; 0.005 g/L NaCl; 0.1 g/L CaCl_2_∙2 H_2_O). The media components were autoclaved separately at 121 °C for 20 min. The initial pH value was adjusted to pH 4 (unbuffered). The *C. aeruginascens* inoculum had a concentration of 0.1 g/L initial dried biomass. 100 µL of this inoculum was added to each well. The cultivation was prepared as a threefold determination for 14 days on a shaker (KS 4000 i control, IKA^®^-Werke GmbH & Co. KG, Staufen, Germany) at 160 rpm and 22 °C. As a reference, 5% orange juice (Sonniger^®^, 100% fruit content, manufactured for ALDI Nord, Essen, Germany) was used.

### 2.2. Influence of the Carbon/Nitrogen Ratio on the Growth of Chlorociboria aeruginascens

To determine the influence of the carbon/nitrogen ratio on the fungal growth, two batches of 12-well plates were prepared. In one batch the initial D-glucose concentrations were adjusted in 1 g/L intervals from 1 to 10 g/L and the yeast extract was adjusted to 9.5 g/L (approximately 0.89 g_N_/L; ratio CH/TN from 1.1 to 11.2). The culture media contained, besides the glucose and the yeast extract, the mineral solution as mentioned in Section 2.1.

In the second batch the initial glucose concentration was adjusted to 10 g/L and the yeast extract concentration was varied in 0.5 g/L intervals from 1 to 12.5 g/L (ratio CH/TN from 107.2 to 9.0). In addition to the glucose and the yeast extract, each culture medium contained a mineral solution (as mentioned in Section 2.1). The work volume measured 3 mL. The media components were autoclaved separately at 121 °C for 20 min. The initial pH value was adjusted to pH 4 (unbuffered). The *C. aeruginascens* inoculum had a concentration of 0.1 g/L initial dried biomass. Then, 100 µL of this inoculum was added to one well. The cultivation was prepared as a threefold determination for 14 days on a shaker at 160 rpm and 22 °C. As a reference *C. aeruginascens* was cultivated in 5% orange juice.

### 2.3. Experimental Investigation of a Cultivation Strategy for Chlorociboria aeruginascens

The results of the investigations in 12-well plates with the glucose-yeast extract medium were transferred to the next cultivation scale. Therefore, 500 mL shaking flasks with a medium volume of 150 mL were used for batch and fed-batch cultivations of *Chlorociboria aeruginascens*. In batch cultivation, 5% orange juice was used as reference medium. The initial medium consisted of 10 g/L glucose and 0.5 g/L yeast extract as well as 1 mL micronutrient solution (25.0 mg/L ZnSO_4_ 7 H_2_O; 25.0 mg/L FeSO_4_ 7 H_2_O; 5.0 mg/L CuSO_4_ 5 H_2_O; 5.0 mg/L MnSO_4_ 4 H_2_O; 1.0 mg/L CoSO_4_ 7 H_2_O; 1.0 mg/L H_3_BO_3_; 0.5 mg/L Na_2_MoO_4_ 2 H_2_O; 0.5 mg/L NiSO_4_ 6 H_2_O; 0.5 mg/L KI). Moreover, 1 mL mineral solution (as described in Section 2.1) was used for a batch and fed-batch cultivation. For the fed-batch cultivation 5 mL yeast extract as a nitrogen source with the concentrations of 3 g/L at day 4, 6 g/L at day 7, 9 g/L at day 9 and 12 g/L at day 11 was fed during the cultivation. The fed nitrogen source was calculated by the autocatalytic reactions for nitrogen limitation with a growth rate µ = 0.31 d^−1^ [20].

For medium preparation each medium ingredient was autoclaved separately at 121 °C for 15 min. Each medium and cultivation strategy was tested as a double determination. The initial dried biomass was measured to 0.19 g/L. Two samples each 2.5 mL for each flask were taken on day 4, 7, 9, 11 (two flasks for each cultivation strategy and two sample each; *n* = 4). The flasks were shaken at 160 rpm (KS 4000 i control, IKA^®^-Werke GmbH & Co. KG, Staufen, Germany) and 22 °C.

### 2.4. Influence of Different Orange Juice Concentrations on the Growth of Chlorociboria aeruginascens

To determine the influence of the initial concentration of orange juice on the growth and pigmentation of *C. aeruginascens*, the fungus was cultivated in 12-well plates with the initial orange juice concentrations of 5%, 10%, 20%, 25%, 30%, 35%, 40% and 50%. The work volume measured 3 mL. The media components were autoclaved separately at 121 °C for 20 min. The *C. aeruginascens* inoculum had a concentration of 0.1 g/L initial dried biomass. Then, 100 µL of this inoculum was added to one well. The cultivation was prepared as a threefold determination for 14 days on a shaker at 160 rpm and 22 °C.

### 2.5. Analyses of Biomass, Sugar and Nitrogen Concentration as well as Xylindein Presence

The dried biomass concentration was determined by gravimetric measuring. Thus, glass microfiber filters (0.7 µm, VWR International) were dried at 60 °C for two days and weighed. The dried filters were inserted in a Reusable Bottle Top Filter (Nalgene™, Thermo Scientific, Massachusetts, USA). For the experimental set-up in 12-well plates, the content (biomass + medium) of all three replicates for each tested nutrient variation was transferred using a pipette with a trimmed tip on the filter. The liquid permeate was stored for further analyses. The filtrate on the glass microfiber filter was washed with 10 mL distilled water and afterwards dried at 60 °C to constant mass. The biomass concentration was calculated by the difference of the biomass loaded filter and empty filter related to the cultivation volume.

The liquid medium at the beginning and the end (day 14) of cultivation were used to determine the concentration of reduced sugars. The concentration of reduced sugars was measured by the 3,5-dinitrosalicylic acid (DNS) method of Miller [21] using d-glucose (4 g/L) as standard, and calculated with the corresponding calibration curve. The absorbance was measured at 545 nm with a GENios Multifunction Microplate Reader (Tecan Group, Männedorf, Switzerland).

To determine the nitrogen concentration, the test kit LCK 238 LATON (HACH LANGE GMBH, Düsseldorf, Germany) was used on instruction [22]. The spectrophotometer Hach Lange DR 2800 (HACH LANGE GMBH, Düsseldorf, Germany) was used for measuring. The measurement of nitrogen content using the test kit is based on the EN ISO 11905-1 [23] and the Koroleff-method.

To determine the presence of xylindein in the filtrated culture medium, the absorbance spectrum (from 250 to 800 nm) of the growth media was measured at the end of cultivation by using a spectrometer (DU 640, Beckman Coulter, Indianapolis, USA). The presence of xylindein in the filtrated culture medium was determined by the detection of the two xylindein typical peaks between 600 nm and 700 nm as reported in [5,6,24].

## 3. Results and Discussion

### 3.1. Influence of Different Carbon and Nitrogen Sources on the Growth of Chlorociboria aeruginascens

As reported in previous publications [9,10], where different substrates were tested as nutrients for *C. aeruginascens*, 5% orange juice was determined to encourage growth and pigment production. From the preceding analysis, the concentration of glucose (1.54 ± 0.2 g/L), sucrose (0.94 ± 0.1 g/L) and fructose (1.78 ± 0.1 g/L) in 5% orange juice were detected. The total concentration of reduced sugar in 5% orange juice amounts to 3.8 ± 0.04 g/L (determined by DNS-method as described in Section 2.5). The total nitrogen content was measured to 39.60 ± 0.1 mg_N_/L and the organic acids was analyzed to 320.5 ± 16.2 mg/L. Five percent orange juice has a natural pH-value of 4 but orange juice is a natural product and therewith its quality is subject to natural fluctuations. To replace orange juice as a culture medium and to understand the metabolism of *C. aeruginascens* and as a result to optimize the growth and xylindein production, various sugars as carbon sources as well as different nitrogen sources were tested according to their influence on growth and pigmentation.

In our previous publication [9] we tested the influence of various nutrients on the growth of *C. aeruginascens*. Different variations of the Kirk’s medium [25] were used where di-ammonium tartrate was applied as a nitrogen source in various concentrations as well as replacing it with urea. It was reported that *C. aeruginascens* did not show any growth in any variations of Kirk’s media. A possible explanation for this reaction might be that the main nitrogen source of Kirk’s medium could not be used by *C. aeruginascens*. Furthermore, it was reported that urea inhibits the growth [9] however, it was not identified whether the high nitrogen content or rather the molecule itself inhibits the growth.

For that reason, three other nitrogen sources (ammonium chloride, nicotinic acid and yeast extract) were tested in combination with different carbohydrates. In general, only in the media with yeast extract was significant fungal growth and pigmentation detected. Media with ammonium chloride and nicotinic acid showed only a small increase in biomass and xylindein production.

For yeast extract and nicotinic acid containing media, xylindein diffusion was documented by measuring the absorption spectrum of the liquid culture media, where the two for xylindein typical absorption peaks at 643 and 700 nm [5,6,9,24] occurred. We have seen various data from literature were the authors used the LAB-method to determine the color shift [5]. In our laboratories we only are able to measure the LAB values in solid media, not in liquid media (filtrated culture medium at the end of cultivation). In most of the literature related to *Chlorociboria* sp.-cultivation where the LAB method was used only the deltaE value is presented. This value is not presenting a color, it is presenting a color shift but not in which color direction. The a and b values would be more interesting to really compare the colors in the blue-green range. Furthermore, LAB is not suitable for measuring the production parameter like intensity or yield. Therefore, we have used the spectrometry. In preparatory work, we were able to make a calibration with extracted xylindein and we were able to prove this with the absorbance spectrum. Hence, an increase of the absorbance in the wavelength range of 660 nm is a sign for increasing xylindein production. Therefore, a quantification is possible with this method. The problem is that no commercial xylindein as standard is currently available.

A further problem is the location of the pigment. It is mainly located in the biomass (intracellular). To quantify the xylindein production, a cell disruption e.g., with French press followed by an extraction and cleaning is needed. The extraction of the xylindein of each biomass in the 12-well plate experiment presented was not possible, because the produced biomass in such a screening experiment is low and the statistic errors would be not acceptable for a proper quantification.

Regarding the nitrogen source and compared with the results of [9], *C. aeruginascens* used the organic nitrogen sources especially the complex nitrogen source like yeast extract for growth. In his experiments, Frenzel [18] also observed a low growth of *Chlorociboria aeruginosum* in inorganic media with different carbohydrate sources. Besides some carbohydrates and fatty acids, yeast extract consists mainly of many proteins, peptides and amino acids as well as vitamins, especially B-vitamins, with nicotinic acid as the main vitamin [26]. Therefore, yeast extract is a good source for the formation of enzymes, its own fungal proteins or to build the nitrogenous cell wall molecule chitin. Even in Stange et al. [9], the reported Kirk’s medium contains low concentration of yeast extract (0.03 g/L instead of the min. 0.1 g/L) used here, which showed no significant influence on the growth. In addition, nicotinic acid; a main vitamin of yeast extract, was tested alone. Contrary to expectation, however, it was unsuitable for supporting fungal growth.

Even when no biomass growth was detected, during the cultivation with ammonium chloride and nicotinic acid with several sugars (all tested except for fructose), the blue-green color of inoculated mycelium became more intense and the color production started earlier (approx. day 5–7) compared to media with the yeast extract (approx. day 9). It means that the fungal metabolism was still active, but not affecting the behavior of growth but rather pigmentation, which can be a result of the production of spores as a way of survival within the environment. Tudor et al. [4] described the blue-green encapsulation of the spores occurring as a higher color density in the biomass.

Beside the effect of the nitrogen source, the influence of the carbon sources was also investigated. The carbon source screening was also prepared on 12-well plates with 3 mL in each well as a three-fold determination. For the reasons outlined above, the most significant results showed media with the yeast extract. Thereby, the tested monosaccharides arabinose, fructose and galactose were not used for biomass production with the fungus. The detailed results of the other carbohydrates are listed in Table 1. The tested disaccharide sucrose was only metabolized with the result of low biomass growth. Therefore, it was not used for further optimization experiments. Sucrose consists of two monosaccharides: glucose and fructose. As *C. aeruginascens* did not show any fungal growth on fructose, the low biomass production of sucrose obviously results from the use of the glucose-monomer. This additionally shows that *C. aeruginascens* is able to produce enzymes which break the α,β-1,2-glycosidic bond. The monosaccharide glucose was also tested as a single carbon source. *C. aeruginascens* used the glucose- as well as maltose-, mannose- and xylose-containing media for biomass growth. Thereby, the media containing glucose and mannose showed the highest biomass production as given in Table 1.

### 3.2. Influence of the Carbon/Nitrogen Ratio on the Growth of Chlorociboria aeruginascens

As reported in Section 3.1, glucose (C-source) and yeast extract (N-source) were determined as good nutrient sources for *C. aeruginascens*. To increase the biomass production of the fungus, different ratios of initial glucose concentration and nitrogen concentration were adjusted in the liquid culture. Therefore, the initial concentration of the nitrogen source was not changed for different cultivation batches, but the initial sugar concentration was varied. In the experiment presented here, the nitrogen concentration was adjusted to approximately 0.89 g_N_/L (equates to a yeast extract concentration of 9.5 g/L), and the glucose concentration was varied from 1 up to 10 g/L at the beginning of the experiment.

**Figure 1:**
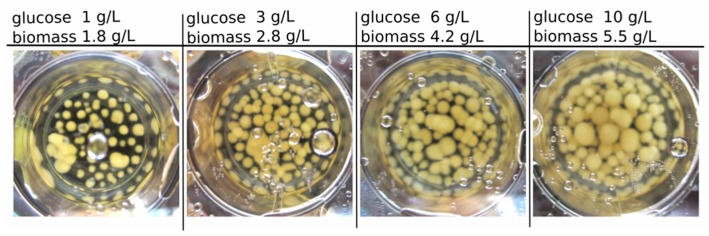
Biomass growth in a 3 mL culture (in 12-well plates) with different initial glucose concentration (1, 3, 6 and 10 g/L) and the final dry biomass concentration at the same initial nitrogen concentration of 0.89 g_N_/L after 14 days of cultivation.

Figure 1 presents exemplary results of the biomass growth of *C. aeruginascens* in 3 mL-culture with different carbohydrate/nitrogen (CH/TN) ratios. The highest dried biomass concentration with 5.5 g/L (after 14 days of cultivation) was identified for the media containing 9.5 g/L yeast extract and 10 g/L glucose (CH/TN ≈ 14) at the beginning of the cultivation.

For all start concentrations, a sugar consumption of 94% ± 3% was investigated. The growth was only dependent on the carbohydrate amount in the media. Therefore, the biomass concentration increased linearly over the substrate concentration (glucose) or CH/TN respectively, as expected.

**Figure 2:**
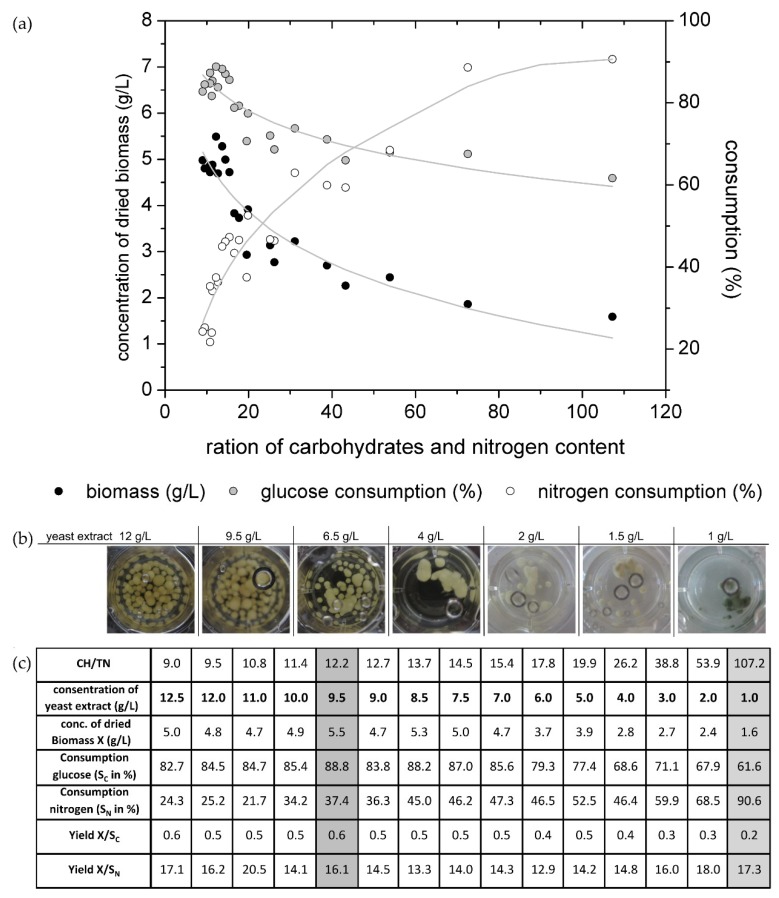
(**a**) Biomass production, glucose and nitrogen consumption of *Chlorociboria aeruginascens* after 14 days of cultivation in 12-well plates with different initial carbohydrate/nitrogen ratios. (**b**) Selected examples of wells with different initial yeast extract contents and different biomass production (well with 1 to 2 g/L are photographed against a white background for better valuation of the pigment production by the fungus). (**c**) Overview of selected data from the cultivation in 10 g/L glucose with variations of yeast extract concentration to different CH/TN ratios, best combination of C and N source for biomass production (marked in dark grey) and best combination for pigmentation (marked in light grey).

No pigmentation was observed for any of the tested media with different initial glucose concentrations. Therefore, the nitrogen content in a liquid culture media with 10 g/L glucose was also verified from 0.1 up to 1.2 g_N_/L (equate 1 to 12.5 g/L yeast extract). Figure 2a shows the biomass production as well as the glucose and nitrogen consumption for the several adjusted CH/TN ratios.

It is well known that several building components like carbon and nitrogen have to be used by an organism for biomass growth. To gain high carbon and high nitrogen contents (low CH/TN ratio) it was expected that the organism would generate high biomass concentrations. As given in Figure 1, for media with low CH/TN (9–15.4) ratios at the beginning of the cultivation, the fungus produced high biomass concentrations, as expected. For those cultivations, the sugar consumption was high while the nitrogen consumption was low, which resulted in the high ability of the nitrogen source. The fungi have an abundance of necessary molecules for biomass growth. Furthermore, it was observed that nearly 90% of the glucose was metabolized by the fungus, but only 50% to 60% were transformed into biomass (yield X/S_C_ is presented in Figure 2c) during the cultivation time. For cultures with high CH/TN ratios, hence with low nitrogen contents, the biomass production was also low. The nitrogen consumption increased but the glucose consumption decreased which resulted in a high abundance of glucose. It is a common effect that microorganisms are not able to metabolize all nutrients when one essential nutrient reaches a critical content. This effect is called growth limitation, caused in this case by the nitrogen source.

The highest dried biomass concentration (with 5.5 g/L) was detected, as mentioned, in the media with 10 g/L glucose and 0.89 g/L (equates to a yeast concentration of 9.5 g/L). In comparison, in the 5% orange juice media the dried biomass concentration after 14 days of cultivation amounts to approx. 1.5 g/L, which is 3.6 times less than that produced with the media tested here. Although, the biomass production of *C. aeruginascens* in the media here is higher than in the orange juice media from [9]. In the orange juice media, the fungus produces xylindein, which has not happened for the media presented here. The effect of rising biomass with the increase of substrate concentration described in [9] was proven as presented in this study. As shown in Figure 2b, the pigmentation of the fungi was only observed in the media with 10 g/L glucose and 1 g/L yeast extract (equate 0.1 g_N_/L nitrogen; CH/TN ≈ 107). For this cultivation 90% of the nitrogen and 61% of glucose was consumed after 14 days. In this experimental set-up the total nitrogen content was determined, which includes also non fungal available nitrogen. Hence, the fungus is not able to consume 100% of the measured nitrogen source. Therewith, the fungal usable nitrogen content in the culture media with 10 g/L glucose and 1 g/L yeast extract reaches its limitation after 14 days of cultivation. The pigment production of the biomass was observed in one replicate on day 12 of this cultivation. On day 14 all replicates showed the blue-green color, which is typical for xylindein. The culture with 1.5 g/L yeast extract (≈0.15 g/L nitrogen) and 10 g/L glucose (CH/TN ≈ 73) also has a high nitrogen consumption and lower glucose consumption. One spot of the biomass started to change its color from white to blue-green at the end of the cultivation (day 14). Probably, if the cultivation time were longer, the biomass would change its complete color to blue-green, due to the limitation of the nitrogen content in the liquid media. For the cultivations with xylindein production, the nitrogen content was nearly completely metabolized, but the media still contained glucose. It was not observed what happens when the nitrogen, as well as the glucose content, decreases into the limitation.

**Figure 3:**
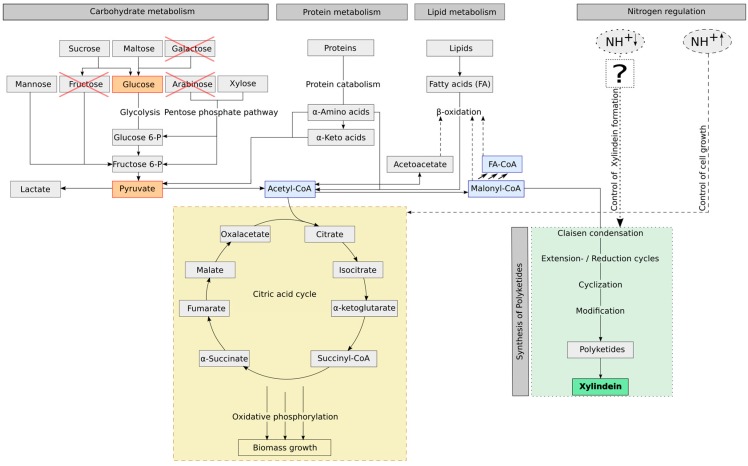
Schematic survey of the influence of various substrate ingredients on the potentially metabolic pathway of *Chlorociboria aeruginascens.* Legend: dotted line—regulation of xylindein formation (green box); dashed line—regulation of biomass growth (yellow box); cross symbol—not metabolized by *C. aeruginascens.*

The fungus produces the pigment in the mycelium (intracellular). From the economic perspective, the biomass and the pigment production of a culture are related. The yield of xylindein in a culture is really low when no biomass is growing. The effect, that *Chlorociboria* sp. also produces pigment in very little biomass, may a result of stress. While the biomass growth is a response of good environmental conditions, the pigment production is related to stress. Hence, for economic reasons it is necessarily to produce a certain amount of stained biomass to extract pigment. As seen in Figure 3, if the fungus has enough nutrients (carbon source as well as nitrogen source), the fungus will produce biomass. When there is a limitation of nitrogen, which is a stress factor for the fungus, it will produce xylindein. However, this will not happen with all nutrient related limitations. Our experiment showed that a glucose limitation is not a regulation factor for the xylindein production. As seen in Figure 2c, the medium with the start concentrations of 9.5 g/L yeast extract and 10 g/L glucose shows a limitation of glucose but no limitation of the nitrogen content. Here, the culture was not producing xylindein (as measured by the absorbance spectrum). In the literature, wood chips were used as an infill of malt extract agar plates [1,4,17]. Hence, wood with its low nitrogen content is a good substrate to force *Chlorociboria* sp. to produce xylindein. When the biomass is low and the intensity of pigmentation is strong, it means that: (A) the ratio of CH/TN has to be high, so there are less molecules, which the fungus can use as carbon source for the biomass growth and a nitrogen limitation appears, which forces the fungus to produce xylindein (nutrient related stress regulation) or (B) there are only few molecules, which can be used for biomass production, but the nitrogen content is high (so CH/TN is low), here there are other environmental conditions like light, pH or eliciting ingredients, which are stress factors for the fungal xylindein production (non-nutrient related stress regulation).

In Figure 3, the influence of substrate ingredients on *Chlorociboria aeruginascens* are summarized and illustrated: (1) the potential metabolic pathway of carbohydrates and nutrients such as yeast extract containing proteins and fatty acids, (2) the influence of the nitrogen source on the pigment production. To understand how nitrogen controls the secondary metabolism was not the aim and thus not investigated in this work. It requires research on the genetic code and gene sequences with relevance for pigmentation.

The production of many fungal secondary metabolites depends on the nitrogen concentration of the growth media. Brzonkalik et al. [27] report that the fungus *Alternaria alternate* produces the mycotoxin Alternariol when the nitrogen concentration in the media is limited. Similar results are presented from Rodriguez-Ortiz et al. [28] for the fungus *Fusarium fujikuroi*, which produces more carotenoids under nitrogen limitation. Further examples are given by Tudzynski [29] for the fungal production of secondary metabolites by nitrogen limitation, but also nitrogen induction. Tudzynski gives a model of the effect of nitrogen limitation on the secondary metabolism of *Fusarium fujikuroi* for production of the red pigment bikaverin. In this model the sufficiency of nitrogen activates TOR (target of rapamycin) a kinase, proliferation, and cell cycle controlling and supporting enzyme. This regulation enzyme is blocked under nitrogen-limiting conditions. However, on the other side, the two GATA transcription factors of filamentous fungi, namely AreA and AreB as nitrogen regulators, are activated and are binding to the promoters of target genes like the bikaverin gen cluster [29]. In addition, the nitrogen-limitation directly affects the expression of the bikaverin gen cluster and therewith the production of the red pigment. Linnemannstöns et al. [30] observed the production of the polyketide synthase in the fungus *Fusarium fujikuroi* under nitrogen-limitation. Gill et al. [12] describe that xylindein is a result of the polyketide synthesis. Hence, this could be a conceivable nitrogen regulation and xylindein production strategy for *Chlorociboria aeruginascens* (as presented as question mark box in Figure 3).

In summary, for media with high nutrient concentrations the biomass production was high and no pigmentation was observed, but for media with low nitrogen concentrations the pigmentation of *C. aeruginascens* occurred, but the biomass production was low. The same effect was observed in [9] for different concentrations of various media. The results presented here show that the nitrogen content is the key for pigmentation of *C. aeruginascens*. The limitation of glucose does not affect the pigment production, but the nitrogen limitation does.

### 3.3. Experimental Investigation of a Cultivation Strategy for Chlorociboria aeruginascens

With these results concerning the nitrogen and carbohydrate source as well as the ratio, a biotechnological strategy for the increase of biomass and induction of the pigmentation was investigated. Therefore, the fed-batch strategy with a stepwise feed of the nitrogen source close to the limitation of the nitrogen content was supposed to increase the yield of xylindein-rich biomass. The nitrogen-based fed-batch strategy with 10 g/L glucose and 0.5 g/L yeast extract as medium was verified by comparison to the batch cultivation with 5% orange juice as medium reference and a nitrogen poor medium for supporting the pigmentation of the fungus (10 g/L glucose and 0.5 g/L yeast extract) in 500 mL shaking flasks (work volume 150 mL).

**Figure 4:**
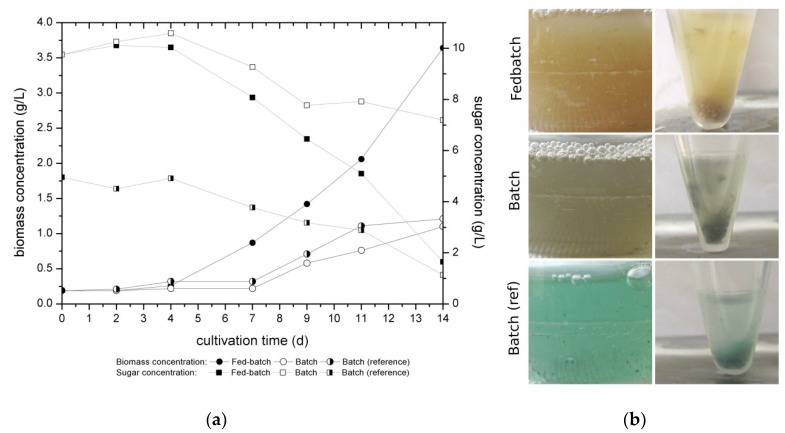
(**a**) Concentration of dried biomass and reduced sugars during the liquid cultivation of *Chlorociboria aeruginascens* as fed-batch (fed at day: 4, 7, 9, 11) and batch cultivation with the initial medium containing 10 g/L glucose and 0.5 g/L yeast extract (defined medium); as reference medium 5% orange juice was used for the batch (reference) cultivation (*n* = 4). (**b**) Appearance of the pigmentation in flask (left column) and of the centrifuged biomass (right column) after 14 days of cultivation.

Figure 4 shows the biomass and sugar concentration of the investigated media in fed-batch and batch cultivation compared with the reference media 5% orange juice as batch cultivation of *C. aeruginascens*.

Thus, *Chlorociboria* sp. belongs to the filamentous fungi; they produce spherical coherent mycelia shapes during liquid cultivation which makes the measuring of the biomass complicated, especially during the first days of cultivation when the biomass concentration is low. Despite this, the results are discussable and fit with previously observed growth behavior of the fungus.

Over the first four days the biomass concentration increased at just a low gradient, which is a common phenomenon called lag-phase where the microorganism is adapting to the new environment. The exponential growth phase starts later for the batch cultivation than for the fed-batch cultivated fungi. On day 4 the first feed with the nitrogen source yeast extract was applied to the fed-batch cultivation, which may be a reason for the increasing biomass concentration. At the same time the nutrients were consumed by the fungus which is represented by the decrease of concentration of reduced sugars (Figure 4a). Regarding the biomass concentration, *C. aeruginascens* shows nearly the same behavior during the batch cultivation with the defined medium and the batch cultivation with the orange juice as a reference medium. The biomass concentration was tripled after 14 days of the fed-batch cultivation compared to the batch strategy. Additionally, the substrate related yield (Y_X/S_) after 14 days of cultivation was 0.41, the highest for the fed-batch cultivation with the defined medium and nitrogen fed (batch with defined medium: Y_X/S_ = 0.32; batch with orange juice: Y_X/S_ = 0.27). While during the fed-batch cultivation 83% (equal 8.1 g/L) and the batch cultivation with 5% orange juice as reference medium, 77% (equal 3.82 g/L) of the reduced sugars were consumed by *C. aeruginascens* in the 14 days of cultivation, only 26% (equal 2.6 g/L) sugar consumption was detected for the batch cultivation with the defined medium caused by the high start concentration of glucose. For the batch cultivation with orange juice the initial concentration of reduced sugars amounted to 4.9 g/L, which is half the sugar amount in comparison to the defined medium (10 g/L). For the batch cultivation with the defined medium, the fungal biomass growth was low. The production of biomass during this cultivation was limited by the low nitrogen content in the medium and therewith, also the consumed reduced sugars were limited. It can be presumed that there is no or just a low increase of real fungal biomass because the fungus needs nitrogen for producing the nitrogen containing chitin for the production of its cell walls. However, for the polyketide pathway and therewith the production of xylindein, *C. aeruginascens* needs Acetyl-CoA, which is formed for instance by the metabolism of sugar (glycolysis) as given in Figure 3. Furthermore, xylindein has a net weight which may be measured as increased biomass growth while no actual increase of cell numbers appears. Additionally, it seems possible that the fungus used the nitrogen of its own dead fungal cells, which are common during filamentous liquid cultivations caused by the formation of mycelia pellets and resulted in an actual increase of the cell numbers. Measuring the cell numbers is not possible for filamentous organisms hence only a presumption can be driven.

Figure 4b gives an impression of the pigmentation appearance of *C. aeruginascens* for the different cultivation strategies and the defined medium and the reference medium. The idea of the fed-batch strategy was to cultivate the fungi near the nitrogen limitation to induce the production of xylindein, but no pigmentation appeared during the cultivation time. Arguably, the fungal culture did not have low enough nitrogen contents, which are necessary for the nitrogen limitation and therewith, the production of xylindein. The pigmentation in the reference medium 5% orange juice displays, as expected of the cultivation, a blue-green discoloration of the biomass and the medium from day 7. The batch cultivation with the defined medium also shows discoloration of the biomass as well as the culture medium which appeared on day 11 of the cultivation. The color differs from the reference medium. The pH-value was measured during the cultivation. The defined medium had an initial pH of 4.8, while the reference medium had an initial pH of 4. Furthermore, the pH increased during the fed-batch cultivation up to 5.9 (after 14 days), while the pH-value of the defined medium during batch cultivation increased at the beginning of the cultivation (until day 4) and decreased (until the end) to a pH-value of 4.9. The reference medium (orange juice) during the cultivation only had a pH-shift of 0.2 by increasing during the cultivation. Possibly the fruit acids in the orange juice have a buffering effect and keep the pH more or less stable. Moreover, the higher pH-value of the defined medium compared to the reference medium may influence the appearance of the discoloration. The influence of the pH-value was also tested and is specified in [19].

To stabilize the pH-value of the defined medium citrate as the buffering substance was added to the new defined medium for batch cultivation of *Chlorociboria aeruginascens*. Furthermore, the glucose concentration regarding the consumption results was adjusted from 10 to 3 g/L. The new defined medium contained 3 g/L glucose, 0.5 g/L yeast extract (approx. 40 mg_N_/L), 0.01 mM citrate, micronutrient solution (as described in Section 2.3) and a mineral solution (as described in Section 2.1). With this medium a great blue-green intensity of discoloration of biomass and medium was generated.

Summarizing the presented results with the presented results in [19], two regulation mechanisms for xylindein production by *C. aeruginascens* were identified. The fungus reacted with the production of xylindein as a result of (A) nitrogen limitation in the substrate and (B) for growth in unfavorable environmental conditions (e.g., light intensity, pH-value) [19].

### 3.4. Influence of Different Orange Juice Concentrations on the Growth of Chlorociboria aeruginascens

In [9] different complex media, e.g., various fruit juices were tested regarding the biomass growth and pigmentation of *C. aeruginascens*. There, 5% orange juice was determined to encourage growth and pigment production of *C. aeruginascens*. Additionally, the correlation of biomass and pigment production with various substrate concentrations was investigated in [9]. Thereby, the substrate was not analyzed regarding the sugar and nitrogen content as well as the consumption.

**Figure 5:**
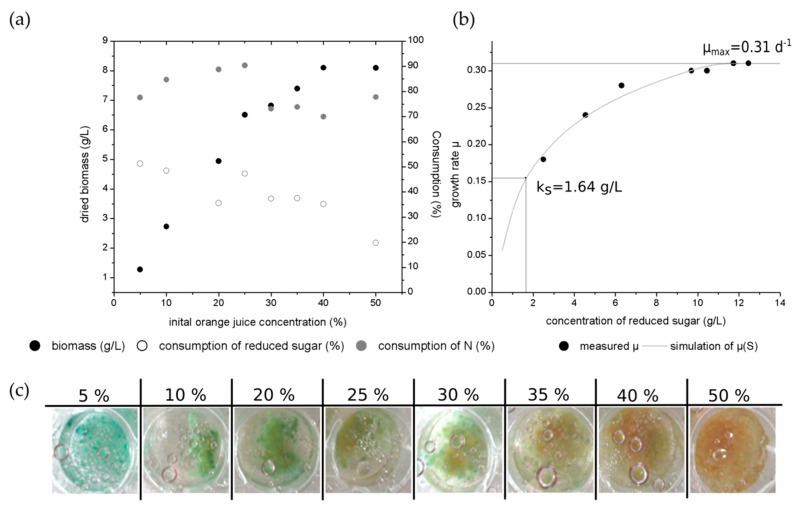
(**a**) Biomass production and consumption of reduced sugars as well as nitrogen in different orange juice concentrations by *Chlorociboria aeruginascens.* (**b**) Measured growth rate µ over the concentration of the consumed reduced sugars as well as calculated half-velocity constant k_S_ and µ_max_ (**c**) Selected examples of wells with different initial orange juice concentrations (5%, 10%, 20%, 25%, 30%, 35%, 40%, 50%).

Figure 5a presents the biomass production as well as the consumption of nitrogen and reduced sugars over the different initial orange juice concentrations. As reported for the increasing glucose concentrations, the highest biomass concentration with 8.1 g/L was observed for 50% initial orange juice concentration.

From the preceding analysis, the sugars composition of 5% orange juice were detected as glucose (1.54 ± 0.2 g/L), sucrose (0.94 ± 0.1 g/L) and fructose (1.78 ± 0.1 g/L). After 14 days of cultivation the entire glucose was consumed. The fructose was not used by *C. aeruginascens*, which proves the reported results in Section 3.1. The sucrose content was measured at the beginning and after 14 days of cultivation with *C. aeruginascens* in 5% orange juice. The fungus used the sucrose as substrate to a lesser extent.

The reduced sugar consumption was measured by DNS-method. Hence, glucose and fructose can be measured with this method. The sucrose is a non-reducing sugar and cannot be detected with the DNS-method. The low consumption of the reduced sugar results in the non-consumed, but measured fructose content. For higher orange juice concentrations, the sugar consumption decreases. Here, cultivation occurs with a high abundance of sugar concentration while not all available fungal sugars were used by *C. aeruginascens*.

The nitrogen consumption is slightly lower than for the cultivations with yeast extract. The explanation for this effect is similar to the sugar consumption. As already reported, *C. aeruginascens* cannot use all nitrogen sources for its growth. Thus, orange juice is a natural product, it consists of several nitrogenous molecules just like proteins, peptides amino acids and vitamins. Hence, the nitrogen content of these molecules is determined by the nitrogen measuring method, but not all of them were used by the fungal culture. For higher initial concentrations of orange juice, there is an oversupply of the available fungal nitrogen sources. As *C. aeruginascens* is a slow growing fungus, not all sugars or nitrogen sources are used for higher initial orange juice concentrations after a cultivation time of 14 days.

Figure 5b presents the growth rate over the different concentrations of reduced sugars. The calculation is based on the MONOD-model, which is used for single-cell organisms. Hence, this model is limited for filamentous organisms as for instance fungal cultures. Thereby, the maximal growth rate (µ_max_) was determined to 0.31 d^−1^ (equal 0.0129 h^−1^) and the half-velocity constant (k_S_) was calculated to 1.64 g/L. By comparison, a maximal growth rate of 0.44 d^−1^ (equal 0.018 h^−1^) was determined for *Trametes hirsuta* in liquid culture [31].

Figure 5c gives an exemplary overview of one well for each used initial orange juice concentration after 14 days of cultivation. The pigmentation occurred on day 9 of the cultivation in 5% orange juice. The pigmentation began later for cultures with higher initial orange juice concentrations (10%—day 11; 20% to 40%—day 13–14). No pigmentation was observed for the culture with 50% orange juice. Thereby, the blue-green color appears with higher intensity for lower orange juice concentrations than for higher initial orange juice concentrations. Furthermore, the blue-green xylindein was also diffused into the liquid media for the initial 5% orange juice concentration. Here, the fungal usable sugars (like glucose and sucrose) or nitrogen sources have a lower concentration at the beginning of the cultivation and hence, they are earlier consumed by the fungus than for the higher initial concentrations. Thus, the fungal culture falls into the limitation of usable nitrogen and sugar sources and reacts by producing the pigment xylindein. For higher initial source concentration, the limitation occurs later than those with lower initial concentrations.

In all further experiments that are presented in this study, 5% orange juice was used to determine the influence of various parameters on the pigmentation of *C. aeruginascens*.

## 4. Conclusions

The studies presented were performed to evaluate the influence of different nutrients on the biomass and pigment production of *Chlorociboria aeruginascens* IHIA39. Therefore, various carbohydrates and nitrogen sources as well as ratios were tested as growth media in liquid culture.

Two important metabolic pathways of the fungus could be observed. The primary metabolic pathway in which uncolored biomass was formed while glucose, mannose, maltose and xylose were used for the fungus as a carbohydrate source. For the investigations presented, the fungus preferred the complex organic nitrogen source yeast extract. For high glucose and high nitrogen concentrations, good biomass growth was observed. The blue-green pigment xylindein was produced in the secondary metabolic pathway caused by a nitrogen limitation in the medium.

With the fed-batch cultivation of *C. aeruginascens*, fed with yeast extract, the biomass production could be increased. However, no pigment was produced. The production of xylindein with defined medium was only successful during batch-cultivation; established by buffering the pH-value with 0.01 mM citrate.

To clarify the fungal growth and to draw a conclusion about the metabolic reaction of the fungus further controlled cultivations with online-measurement of various process parameters as well as biomass and substrate monitoring are proposed.

## Figures and Tables

**Table 1:** Dried biomass concentration for different culture media containing various carbohydrates (1 g/L) and yeast extract (0.1 g/L).

Carbohydrate	Glucose	Maltose	Mannose	Sucrose	Xylose
**Dried biomass**	0.21 g/L	0.17 g/L	0.28 g/L	0.05 g/L	0.15 g/L
